# *N*-AC-l-Leu-PEI-mediated miR-34a delivery improves osteogenic differentiation under orthodontic force

**DOI:** 10.18632/oncotarget.22790

**Published:** 2017-11-30

**Authors:** Wenwen Yu, Yi Zheng, Zhujun Yang, Hongbo Fei, Yang Wang, Xu Hou, Xinhua Sun, Yuqin Shen

**Affiliations:** ^1^ Department of Orthodontics, School and Hospital of Stomatology, Jilin University, Changchun 130021, China; ^2^ Department of Periodontics, School and Hospital of Stomatology, Jilin University, Changchun 130021, China

**Keywords:** miR-34a, osteogenic differentiation, orthodontic tooth movement, strain, N-acetyl-L-leucine-polyethylenimine

## Abstract

Rare therapeutic genes or agents are reported to control orthodontic bone remodeling. MicroRNAs have recently been associated with bone metabolism. Here, we report the *in vitro* and *in vivo* effects of miR-34a on osteogenic differentiation under orthodontic force using an *N*-acetyl-L-leucine-modified polyethylenimine (*N*-Ac-l-Leu-PEI) carrier. *N*-Ac-l-Leu-PEI exhibited low cytotoxicity and high miR-34a transfection efficiency in rat bone mineral stem cells and local alveolar bone tissue. After transfection, miR-34a enhanced the osteogenic differentiation of *Runx2* and *ColI*, Runx2 and ColI protein levels, and early osteogenesis function under orthodontic strain *in vitro*. MiR-34a also enhanced alveolar bone remodeling under orthodontic force *in vivo*, as evidenced by elevated gene and protein expression, upregulated indices of alveolar bone anabolism, and diminished tooth movement. We determined that the mechanism miR-34a in osteogenesis under orthodontic force may be associated with GSK-3β. These results suggested that miR-34a delivered by *N*-Ac-l-Leu-PEI could be a potential therapeutic target for orthodontic treatment.

## INTRODUCTION

Despite advances in orthodontic appliances and techniques, poor alveolar bone condition remains challenging in orthodontic practice. Complex alveolar bone conditions, including bone absorption caused by periodontitis and congenital degradation resulting from high-angle malocclusion, can lead to uncontrollable movement of anchorage teeth that negatively affect treatment outcomes. Improving the local alveolar bone formation is crucial for controlling tooth movement. The differential expression of microRNA (miRNA) during odontogenesis [1–2], tooth development [3–5], and tooth movement [6–10] was recently reported. MicroRNA-34a (miR-34a) was expressed in multiple stages of tooth growth and regulated bone remolding [11] and dental stem cell differentiation [12–13]. MiR-34a endogenously inhibited osteoclasts that block osteoporosis and bone metastasis by suppressing osteoclastogenesis and TGIF2 expression. MiR-34a also regulated the osteogenesis process of osteosarcoma inversion to osteogenic differentiation [14]. However, miR-34a did not enhance osteogenesis enough to attenuate systemic bone loss in OVX mice or bone cancer metastases [11]. Despite insufficient regulation of systemic bone formation [11], miR-34a could attenuate local bone loss or alveolar bone remodeling to achieve stability of anchorage teeth. We established an *in vitro* model of bone marrow stem cells (BMSCs) under mechanical force and an *in vivo* model of orthodontic tooth movement (OTM) in rats to investigate miR-34a as a local therapeutic target of orthodontic treatment.

MiRNAs do not readily cross the cell membrane to reach target genes because of their negative charge, degradability, and inherent instability. Delivery systems must be applied to accomplish local gene delivery. Non-viral carriers, such as the polymer-mediated delivery system of branched PEI (25 kDa, PEI25K), have demonstrated efficient gene transfection both *in vivo* and *in vitro* [15–19]. However, the positive charge of PEI25K caused cytotoxicity, hemolysis, and serum instability from cell membrane breakage and nonspecific interactions with negatively charged serum proteins [20–21]. PEI25K was chemically modified by hydrophobic group or polymer grafting to enhance the transfection efficiency and decrease cytotoxicity. The *N*-Ac-l-Leu-PEI derivative was previously constructed by grafting hydrophobic *N*-acetyl-L-leucine on PEI25K. *N*-Ac-l-Leu-PEI demonstrated superior cell viability, biocompatibility, and gene release rates compared to PEI25K [22]. *N*-Ac-l-Leu-PEI is a functional, biocompatible vehicle for gene delivery *in vitro* and *in vivo*. For example, this carrier successfully delivered the p53 gene [22] and DNAzyme [23] and inhibited tumor cell proliferation and migration. Our study is the first known example of miRNA delivered by *N*-Ac-l-Leu-PEI to rBMSCs and local alveolar bone.

The effect of miR-34a on osteogenesis under orthodontic force was systematically evaluated *in vitro* and *in vivo*. The binding affinity, biocompatibility, and delivery capacity of *N*-Ac-l-Leu-PEI as a miR-34a carrier were investigated. Marker gene and protein expression were analyzed *in vitro* to determine the effect of miR-34a on early functions of osteogenic differentiation under orthodontic force. The effect of miR-34a on local alveolar bone remodeling and tooth movement *in vivo* was investigated by qRT-PCR, morphology observations, and micro-CT assay.

## RESULTS

### MiR-34a regulation during orthodontic alveolar bone remodeling

**Figure 1:**
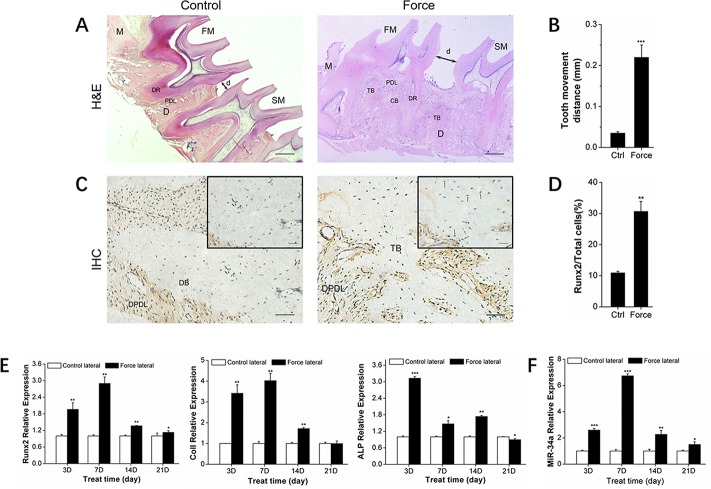
Osteogenesis and miR-34a expression of alveolar bone under orthodontic force loading **(A)** H&E staining showed the morphological characteristics and distance of tooth movement during orthodontic tooth movement for 14 days. Scale bars: 500 μm. *n*=3. **(B)** Statistical analysis of tooth movement distance. *n*=6. **(C)** Representative immunohistochemical staining showed *Runx2^+^* cells of alveolar bone during orthodontic tooth movement for 14 days. Black arrows marked positively stained dark-brown granules. Scale bars: 100 μm and 20 μm. *n*=3. **(D)** Statistical analysis of *Runx2^+^* signals in images was similar to those shown in Figure 1C. **(E)** qRT-PCR analysis of osteogenic genes, *Runx2, ColI*, and *ALP* during orthodontic tooth movement. *n*=3–4. **(F)** qRT-PCR analysis of miR-34a during orthodontic force loading. *n*=3–4. Data represent the mean ± SD of three independent experiments. ^*^P<0.05 vs. the control lateral bone by paired *t*-test. FM: first molar. SM: second molar. DR: dental root. PDL: periodontal ligament. DPDL: distal PDL. DB: distal bone. TB: tensile lateral bone. CB: compressive lateral bone. M: mesial. D: distal. d: moving distance of FM or the distance between FM and SM.

**Figure 2:**
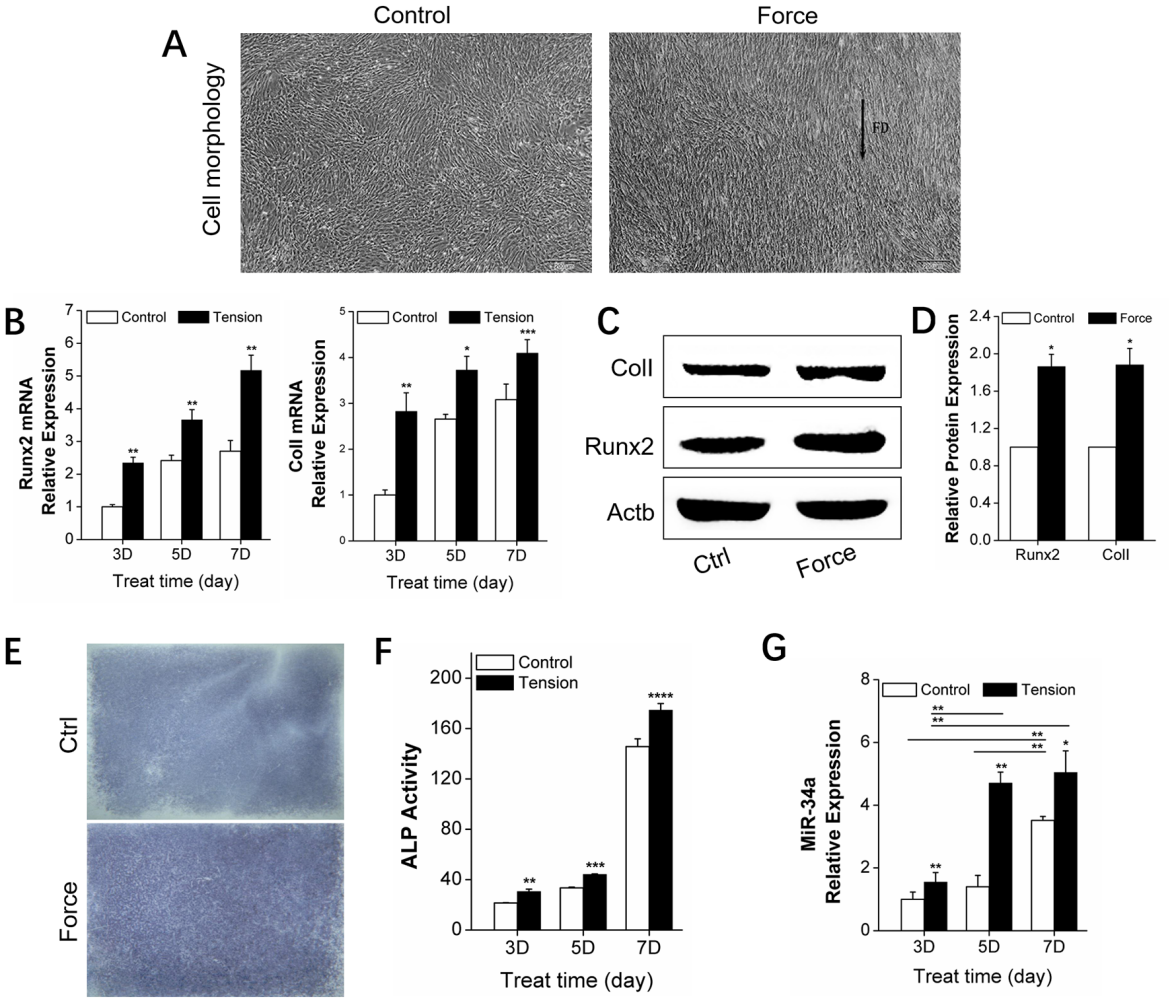
*In vitro* promotion of osteogenic differentiation by orthodontic strain and miR-34a expression in rBMSCs **(A)** rBMSCs under non-tension condition (ctrl group) in the first image and BMSCs under orthodontic force condition (force group) for 5 days in the second image. *n*=4. Scale bars: 200 μm. FD: force direction. **(B)** qRT-PCR analysis of osteogenic genes, *Runx2*, and *ColI*, under orthodontic force condition on day 3, 5, and 7. *n*=3. **(C** and **D)** Western blot analysis (quality in C and quantity in D) of Runx2 and ColI protein levels of the control and force groups on day 7. *n*=3. **(E)** ALP staining of rBMSCs in the control and force groups after 7 days. *n*=3. ALP: alkaline phosphatase. **(F)** ALP activity analysis of rBMSCs under force conditions on day 3, 5, and 7. *n*=3. **(G)** qRT-PCR analysis of miR-34a expression under force conditions on day 3, 5, and 7. *n*=3. Data represent the mean ± SD of three independent experiments. ^*^P<0.05 vs. the control group by paired t-test.

Alveolar bone remodeling is considered the biological basis of orthodontic tooth movement (OTM) [24–25]. MiRNAs were recently determined to be key bone metabolism regulators. Specifically, miR-34a was previously demonstrated as an important factor in bone metabolism in phase I clinical trials [26]. We established an *in vivo* unilateral OTM model in rats and an *in vitro* model of rBMSCs bearing force loading to investigate the miR-34a regulation of orthodontic-mediated alveolar bone remodeling. The *in vivo* OTM model was successfully established (Figure 1A–1B). The expression of the bone-specific protein, Runx2, was increased (Figure 1C–1D) during this process. *Runx2, ColI*, and *ALP* bone differentiation genes showed elevated expression (Figure 1E). After force loading *in vitro*, the rBMSCs aligned along the force direction (Figure 2A). Strain force also improved osteogenic differentiation (Figure 2B–2F). MiR-34a expression was upregulated and reached a maximum at day 7 under orthodontic force loading (Figures 1F and 2G) *in vivo* and *in vitro*. The variation in miR-34a expression was consistent that of osteogenic differentiation factors. Therefore, we selected miR-34a as a model miRNA to investigate the regulation of bone remodeling during orthodontic force loading.

### Functional evaluation of *N*-Ac-l-Leu-PEI carrying miR-34a *in vitro*

We determined the effect of miR-34a on the strain-induced bone formation and the safety and efficiency of the *N*-Ac-l-Leu-PEI delivery system. Orthodontic patients are unfit for scaffold implantation or intravenous injection, similar to patients with bone defects or osteoporosis. We used *N*-Ac-l-Leu-PEI as a carrier to overcome the limitations of injection-based miR-34a delivery.

**Figure 3:**
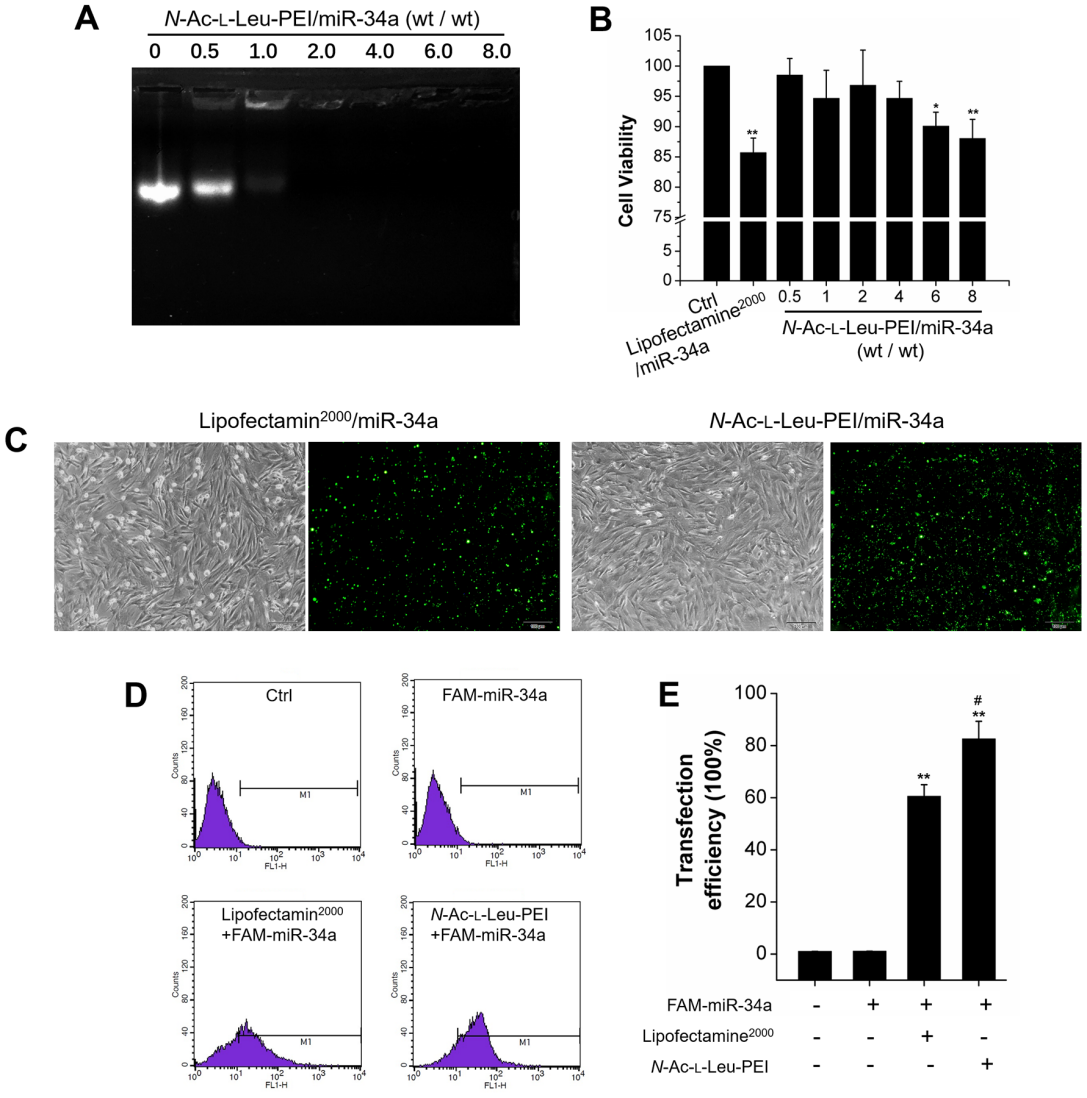
Functional evaluation of *N*-Ac-l-Leu-PEI/miR-34a **(A)** Gel retardation assay for *N*-Ac-l-Leu-PEI complexes with miR-34a at different mass ratios. *n*=3. **(B)** Cell viability of rBMSCs treated with Lipofectamin^2000^/miR-34a or *N*-Ac-l-Leu-PEI/miR-34a complexes with various mass ratios for 24 h. *n*=3. **(C)** Microscopic images of FAM-miR-34a-positive rBMSCs under ordinary light and blue light. Scale bar: 100 μm. *n*=3. **(D)** Flow cytometry analysis of FAM-positive rBMSCs. *n*=3. **(E)** Statistical analysis of FAM-miR-34a-positive signals in images similar to those shown in Figure 3D. *n*=3. Data represent the mean ± SD of three independent experiments. ^*^P<0.05 vs. the control group by paired *t*-test.

An agarose gel retardation assay was used to detect the binding affinity of *N*-Ac-l-Leu-PEI and miR-34a. The condensing capacity of *N*-Ac-l-Leu-PEI improved in the presence of miR-34a. *N*-Ac-l-Leu-PEI and miR-34a formed a stable nanocomplex with a mass ratio of 2 (Figure 3A). MTT assay determined the biocompatibility of *N*-Ac-l-Leu-PEI. The cell viability of *N*-Ac-l-Leu-PEI groups was superior to Lipofectamine^2000^ groups and exceeded 85% at a mass ratio of 8 (8 μg/mL) (Figure 3B). Fluorescence microscopy and flow cytometry were used to detect the transfection capacity of *N*-Ac-l-Leu-PEI. The transfection efficiency of *N*-Ac-l-Leu-PEI and Lipofectamine^2000^ in rBMSCs was estimated to be 70–80% and 50–60%, respectively, from the percentage of FAM-miR-34a positive cells observed by inverted fluorescence microscopy (Figure 3C). Flow cytometry quantified the transfection efficiency and *N*-Ac-l-Leu-PEI was more efficient than Lipofectamine^2000^ (Figure 3D–3E). We concluded that *N*-Ac-l-Leu-PEI exhibited superior biocompatibility and higher transfection efficiency compared to Lipofectamine^2000^ with adequate condensing capacity as a miR-34a delivery vehicle.

### MiR-34a regulation of osteogenic differentiation under orthodontic strain *in vitro*

**Figure 4:**
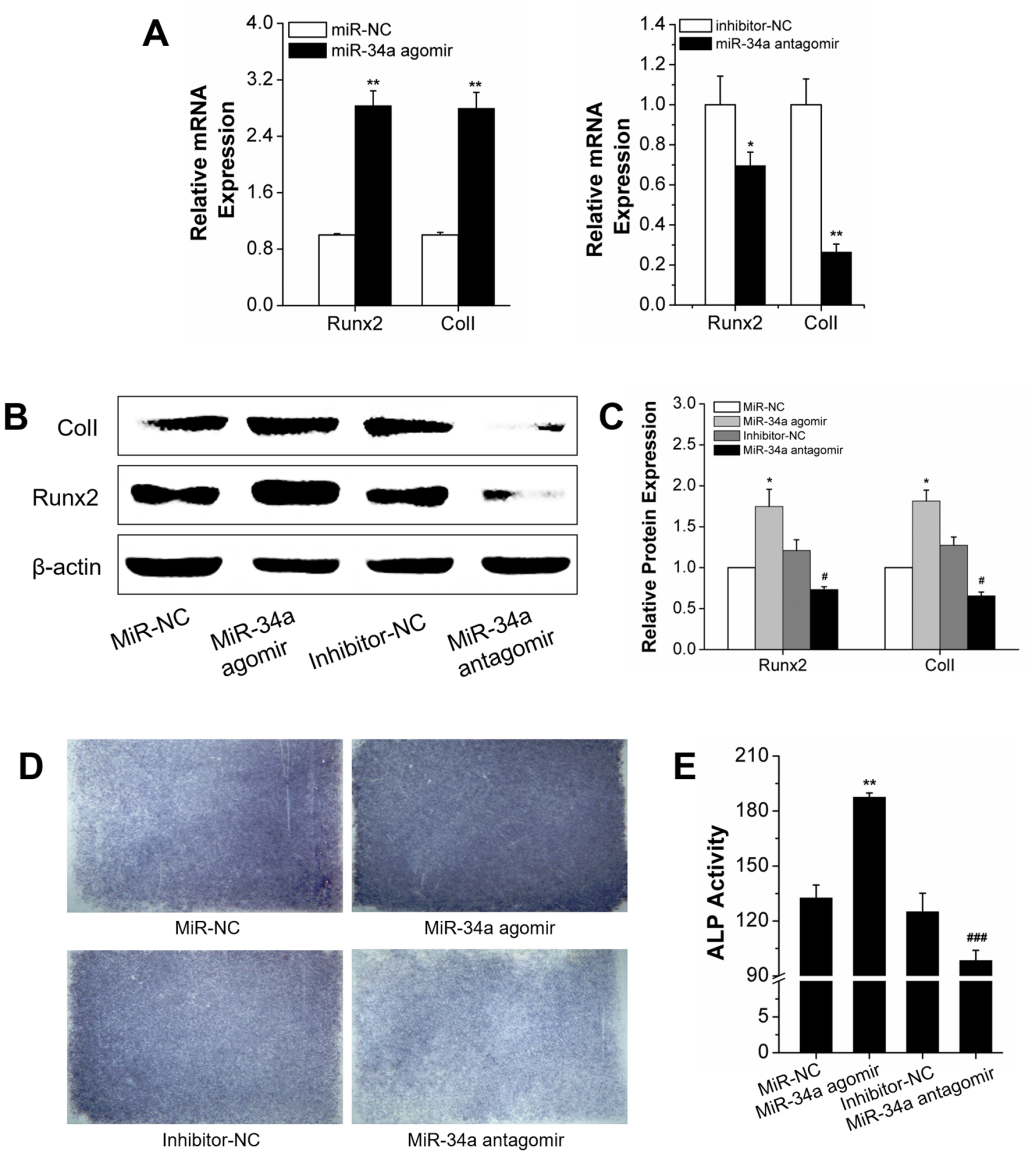
Osteogenic differentiation under orthodontic strain was improved by the MiR-34a agomir *in vitro* **(A)** Quantitative real-time PCR analysis of Runx2 and ColI mRNA expression of agomir transfected rBMSCs for 7 days. *n*=3. **(B** and **C)** Western blot analysis (quality in C and quantity in D) of Runx2 and ColI protein levels of miR-34a agomir and antagomir transfected rBMSCs on day 7. *n*=3. **(D)** ALP staining of miR-34a transfected rBMSCs for 7 days. *n*=3. **(E)** ALP activity analysis of miR-34a transfected rBMSCs for 7 days. *n*=3. Data represent the means ± SD of three independent experiments. ^*^P<0.05 vs. the miR-NC strain group by paired *t*-test, ^#^P<0.05 vs. the inhibitor-NC strain group by paired *t*-test.

We determined miR-34a regulation of osteogenic differentiation under orthodontic force loading. The orthodontic force loading model was built with a four-point bending system [27] to determine the effect of miR-34a on osteogenesis under force loading *in vitro*. After transfection of miR-34a agomir or antagomir, the rBMSCs with *N*-Ac-l-Leu-PEI/miR-negative control (NC) and the inhibitor-NC control group were subjected to an orthodontic strain force. MiR-34a regulation of strain-induced osteogenesis was determined by gene and protein expression and early secretion function. The qRT-PCR results indicated that the miR-34a agomir induced *Runx2* and *ColI* gene expression. The miR-34a antagomir had an opposite effect on gene expression compared to inhibitor-NC (Figure 4A). Protein expression (Runx2 and ColI) was consistent with the respective gene expression (Figure 4B–4C). ALP activity is an early characteristic of osteoblast differentiation. MiR-34a agomir elevated ALP expression and activity (Figure 4D–4E) after exposure to force loading conditions for 7 days. We concluded that miR-34a had a positive effect on osteogenic differentiation under force loading conditions *in vitro*.

### Transfection efficiency and biocompatibility of *N*-Ac-l-Leu-PEI-mediated miR-34a delivery *in vivo*

**Figure 5:**
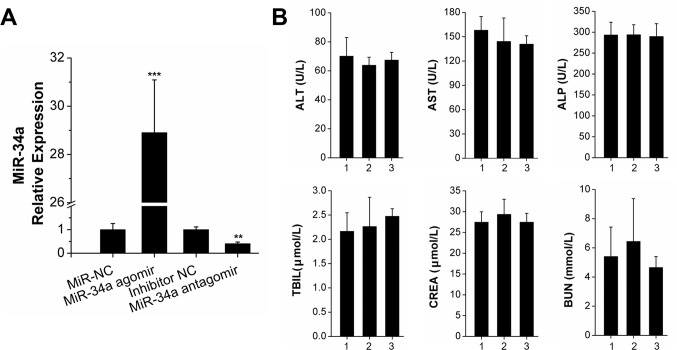
*N*-Ac-l-Leu-PEI-mediated miR-34a delivery led to high local transfection efficiency and compatibility *in vivo* **(A)** qRT-PCR analysis of *N*-Ac-l-Leu-PEI carrying miR-34a during OTM for 14 days. *n*=3. **(B)** The detection of serum biochemical indices. The numbers 1, 2, 3 represented the control group, miR-34a agomir group, miR-34a antagomir group, respectively. *n*=3–4. Data represent the mean ± SD of three independent experiments. ^*^P<0.05 vs. the miR-NC lateral bone for Figure 5A and control group for Figure 5B by paired *t*-test.

The transfection efficiency and serum biocompatibility of *N*-Ac-l-Leu-PEI-mediated miR-34a delivery *in vivo* were determined to account for differences between *in vitro* and *in vivo* microenvironments. We established a bilateral tooth movement model in rats to investigate the local expression of miR-34a delivered by *N*-Ac-l-Leu-PEI. The expression in separate bilateral alveolar bones was determined by qRT-PCR assay after 14 days. An injection of 2 μg/time/lateral ensured local expression of miR-34a in the experimental area of the alveolar bone. The expression level of miR-34a was approximately 29-fold with miR-34 agomir and 0.4-fold with miR-34a antagomir (Figure 5A). These results indicated that *N*-Ac-l-Leu-PEI delivered miR-34a locally *in vivo*.

Immune-related adverse events of MRX34 (liposomal miR-34a mimic) have been observed over the course of clinical trials [28]. We analyzed the biochemical indices of the successfully transfected rats. The differences between the control, miR-34a agomir, and miR-34a antagomir groups were not statistically significant. We concluded that a small local injection dose of miR-34a did not affect the liver or kidney function of the rats.

### Alveolar bone remodeling by *N*-Ac-l-Leu-PEI-mediated miR-34a delivery *in vivo*

**Figure 6:**
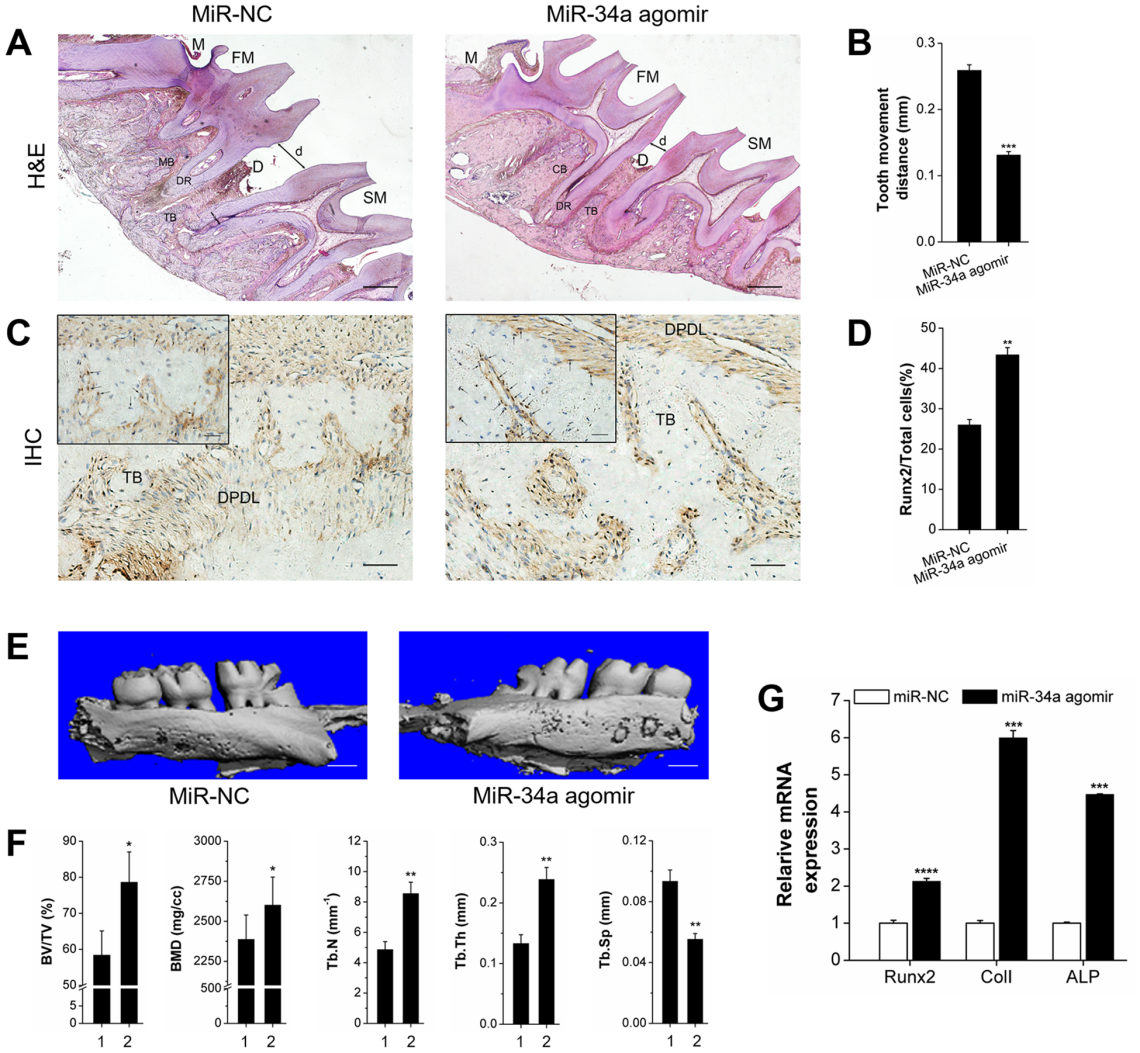
Osteogenesis analysis of OTM with local miR-NC or miR-34a agomir application *in vivo* **(A)** H&E staining showed the morphological characteristics and distance of tooth movement with local application of miR-34a agomir during orthodontic tooth movement for 14 days. Scale bars: 500 μm. *n*=3. **(B)** Statistical analysis of tooth movement distance. *n*=6. **(C)** Representative immunohistochemical staining showed *Runx2^+^* cells of alveolar bone with local application of miR-34a agomir during orthodontic tooth movement for 14 days. Black arrows marked positively stained dark-brown granules. Scale bars: 100 μm and 20 μm. *n*=3. **(D)** Statistical analysis of *Runx2^+^* signals in images similar to those shown in Figure 6C. **(E)** Three-dimensional images of the tooth movement and local alveolar bone for 14 days were tested by micro-CT. Scale bars: 1 mm. *n*=3. **(F)** Quantitative analysis of BV/TV, BMD, Tb.N, Tb.Th, and Tb.Sp. *n*=3. 1 and 2 presented the miR-NC lateral and miR-34a agomir lateral. **(G)** qRT-PCR analysis of osteogenic genes, *Runx2, ColI*, and *ALP* on miR-NC and miR-34a agomir lateral bone during OTM for 14 days. *n*=3. Data represent the mean ± SD of three independent experiments. ^*^P<0.05 vs. the miR-NC lateral bone by paired *t*-test.

**Figure 7:**
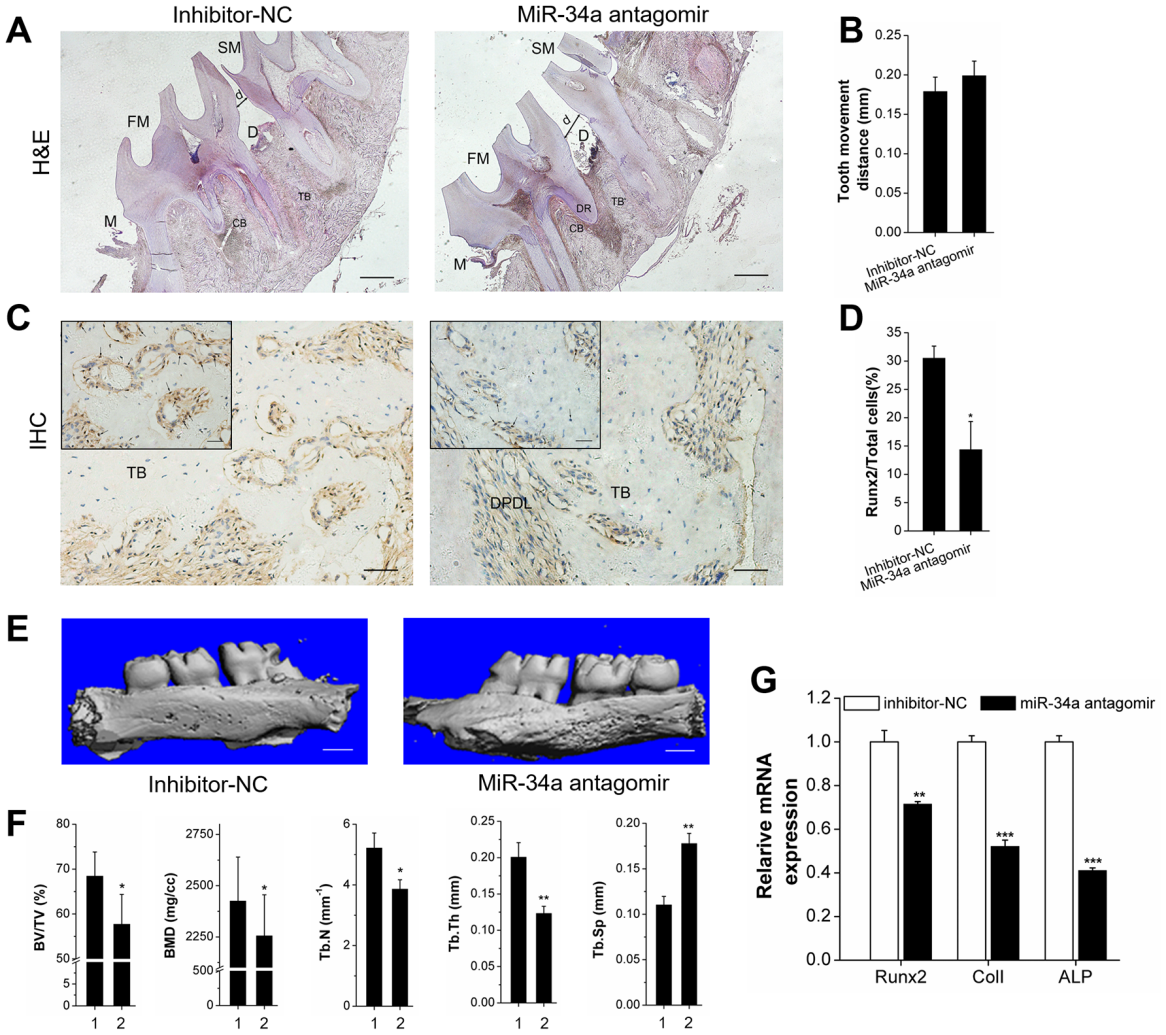
Declining osteogenesis of local *N*-Ac-l-Leu-PEI-mediated miR-34a antagomir delivery on alveolar bone and OTM *in vivo* **(A-E)** The alveolar bone of rats was locally injected with *N*-Ac-l-Leu-PEI/miR-34a antagomir (and inhibitor-NC), and the legends of Figure 7A–7G corresponded to those from Figure 6 **(A**–**G)**. 1 and 2 presented the inhibitor-NC lateral and miR-34a antagomir lateral. Data represent the mean ± SD of three independent experiments. ^*^P<0.05 vs. the inhibitor-NC lateral bone by paired *t*-test.

We investigated the effect of miR-34a on local alveolar bone remodeling and tooth movement. Morphology characterization, micro-CT assay, and qRT-PCR were used. The OTM distance was decreased by lateral bone miR-34a agomir delivery compared with that of lateral bone miR-NC (Figure 6A, 6B, 6E). The stained granule density of *Runx2*^+^ cells was increased (Figure 6C and 6D). Furthermore, *Runx2*^+^ cells were distributed in the marrow cavity of the alveolar bone. The elevated fullness and height of buccal alveolar bone were concentrated around the first molar (Figure 6E). The bone anabolism indices (BV/TV, BMD, Tb.N, and Tb.Sp; Figure 6F) were improved, and the *Runx2, ColI*, and *ALP* gene expression was upregulated (Figure 6G). However, the improvement of alveolar bone mass was completely reversed by *N*-Ac-l-Leu-PEI/miR-34a antagomir delivery (Figure 7). Though the movement distance of first molar on *N*-Ac-l-Leu-PEI/miR-34a antagomir lateral was not statistically different from that of *N*-Ac-l-Leu-PEI/inhibitor-NC lateral (Figure 7A, 7B, 7E), the fullness and height of the first molar buccal alveolar bone were remarkably reduced according to the micro-CT image (Figure 7E). The *Runx2*^+^ cell density (Figure 7C and 7D), indices of bone anabolism (Figure 7F), and expression of osteogenesis genes (Figure 7G) declined. Therefore, MiR-34a increased osteogenesis and bone formation in force-induced local alveolar bone remodeling and alleviated tooth movement.

### Wnt signal pathway as a potential mechanism of miR-34a-mediated osteogenic differentiation under orthodontic force loading

**Figure 8:**
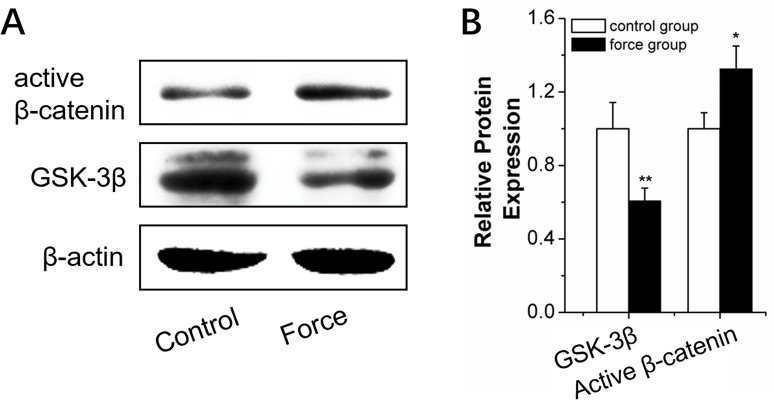
Wnt/β-catenin signal pathway analysis in BMSCs under strain **(A** and **B)** Western blot (quality in A and quantity in B) analysis of Wnt/β-catenin-signal-pathway-related proteins, GSK-3β, and active β-catenin for 2 h under strain loading. *n*=3. Data represent the mean ± SD of three independent experiments. ^*^P<0.05 vs. the control group by paired *t*-test.

We explored the mechanism of miR-34a regulation of force-induced osteogenic differentiation. Genetic software and previous reports [29–30] indicated that many miR-34a target genes in bone metabolism were associated with the Wnt/β-catenin pathway. This pathway is known as the primary mechano-transduction mechanism of bone remodeling [31–32]. Early, rapid, transient β-catenin response and GSK-3β inhibition were observed after orthodontic force stimulation for 2 hours (Figure 8).

**Figure 9:**
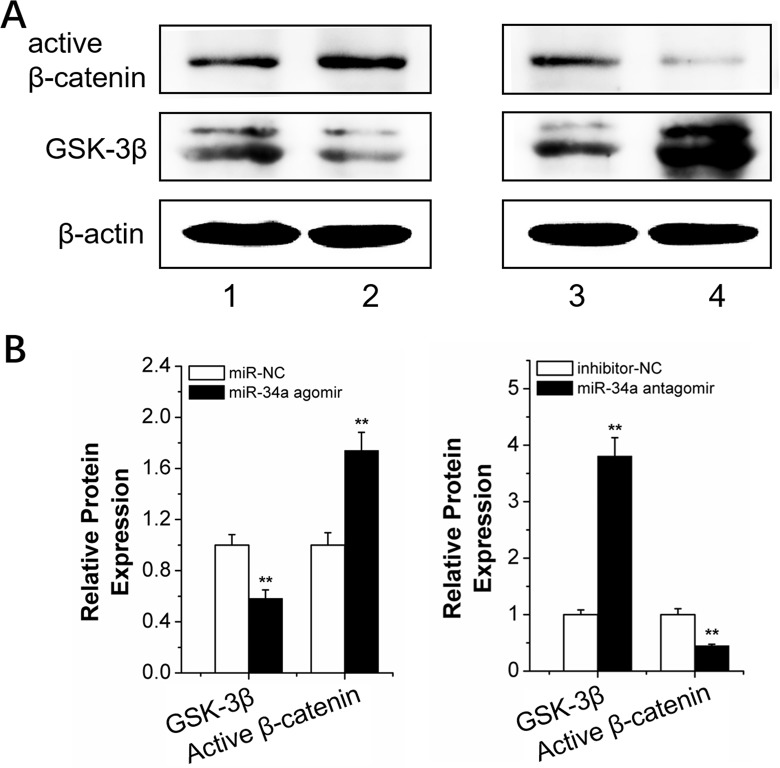
MiR-34a regulation of the Wnt/β-catenin signal pathway **(A** and **B)** Western blot (quality in A and quantity in B) analysis of the Wnt/β-catenin signal pathway proteins, GSK-3β and active β-catenin, after 2 h of strain and transfection with miR-34a. *n*=3. 1, 2, 3, 4 presented miR-NC, miR-34a agomir, inhibitor-NC and miR-34a antagomir. Data represent the means ± SD of three independent experiments. ^*^P<0.05 vs. the miR-NC or inhibitor-NC strain group by paired *t*-test.

We determined the expression of Wnt/β-catenin pathway-related proteins in rBMSCs transfected with *N*-Ac-l-Leu-PEI/miR-34a agomir or antagomir to elucidate the mechanism of miR-34a-mediated osteogenesis during force loading. GSK-3β was downregulated after *N*-Ac-l-Leu-PEI/miR-34a agomir delivery compared to the *N*-Ac-l-Leu-PEI/miR-NC strain group under conditions of orthodontic strain. Active β-catenin, indicative of the active Wnt/β-catenin pathway, was elevated as the GSK-3β level decreased (Figure 9). The miR-34a antagomir had an adverse effect on GSK-3β and active β-catenin expression (Figure 9). Our results suggested that GSK-3β was sensitive to miR-34a under orthodontic strain.

**Figure 10:**
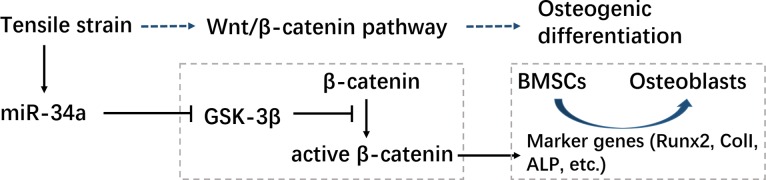
Schematic of the regulatory mechanism of miR-34a in osteogenesis during strength loading “→”: active; “—|”: inhibit.

We propose a potential mechanism for miR-34a in osteogenic differentiation during orthodontic force loading. Force induced miR-34a targets GSK-3β after orthodontic force loading, which downregulates the inhibition of β-catenin protein phosphorylation. Active β-catenin gradually accumulates in the cell nucleus to initiate the Wnt/β-catenin pathway, which activates the downstream genes of osteogenic differentiation, such as *Runx2* (Figure 10) [33].

## DISCUSSION

An understanding of alveolar bone remodeling and tooth movement depend on suitable physiological strain stimulation. The mechanical forces induced by a four-point bending system closely modeled the physiological strain of the clinical loading mode of cells [34]. In our study, physiological strain was associated with improved bone remodeling in moderate agreement with previous studies [27, 35]. MiR-29 [8] and miR-494-3p [9] were also associated with osteogenesis after a mechanical stimulus. MiR-21 inhibited OTM by preventing force- or periodontitis-induced maxillary bone loss [10], which indicated the potential role of miRNA in alveolar bone remodeling during orthodontic treatment. Our study supported miR-34a involvement in tooth development [12, 13], mechanical force loading [36], and regulation of bone metabolism [11, 37–41].

We explored the role of miR-34a delivered by an *N*-Ac-l-Leu-PEI carrier in the regulation of bone remodeling induced by orthodontic force. We demonstrated that miR-34a elevated osteogenic gene and protein expression, early osteogenic secretion function, and local alveolar bone anabolism under orthodontic force loading *in vivo* and *in vitro*. Moreover, miR-34a decreased the OTM. The enhanced osteogenesis *in vitro* from our data was inconsistent with Chen L [38], Wei J [39] and Tamura M [40], who reported that miR-34s and miR-34a inhibited osteogenesis under static conditions. However, the *in vivo* conditions were different from those *in vitro*. We demonstrated that alveolar bone anabolism *in vivo* was stimulated by orthodontic force for 24 h. The extended force loading time enhanced miR-34a-mediated osteogenic stimulation. Improved osteogenesis was also supported by the *in vivo* studies of Krzeszinski [11] and Fan [41]. We demonstrated that miR-34a enhanced force-mediated osteogenic differentiation *in vitro* and *in vivo* and alleviated OTM by increasing local osteogenesis of the alveolar bone.

We selected the PEI-derivative, *N*-Ac-l-Leu-PEI, as a delivery vehicle for miR-34a. *N*-Ac-l-Leu-PEI overcame the low biocompatibility of PEI25K and exhibited low cytotoxicity, which was consistent with the results of Li [22]. These features might be attributed to reduced cationic density and the downregulation of cell membrane breakage. Furthermore, *N*-Ac-l-Leu-PEI had a lower binding affinity for miRNAs and single-stranded oligoDNA (both of their critical mass rate at 2.0 [23]) than for plasmid DNA (critical mass rate of 0.6–0.8 [22]). *N*-Ac-l-Leu-PEI efficiently delivered miR-34a to rBMSCs (≈70%) and alveolar bone (29-fold). These results were superior to those of Krzeszinski [11], who obtained an approximately 5-fold efficiency increase in bone marrow with pre-miR-34a delivered by chitosan nanoparticles after a single injection. We suggest that further pretreatment of miRNAs carried by *N*-Ac-l-Leu-PEI could have the potential for orthodontic therapy in patients with poor alveolar bone conditions.

Understanding the miR-34a mechanism under orthodontic force by grafting target agents on the *N*-Ac-l-Leu-PEI delivery system could lead to optimal specificity and efficiency of miR-34a-mediated bone remodeling. Wnt/β-catenin pathway genes were modified by mechanical force during osteogenesis [42]. Wnt/β-catenin was recently demonstrated as the most important pathway of mechanotransduction and osteogenic differentiation. Our results demonstrated the early, rapid response of the Wnt/β-catenin pathway to mechanical stimulus that enhanced bone sensitivity to mechano-transduction [43]. MiR-34a and the Wnt/β-catenin pathway were closely associated. GO and KEGG analysis revealed that miR-34a expression improved and β-catenin expression increased during bone repair [44]. P53 induced the Wnt/β-catenin pathway by direct miR-34a regulation of Axin2 and GSK-3β [45]. We demonstrated that GSK-3β was the target protein of miR-34a under orthodontic force loading. GSK-3β prevented activation of the Wnt pathway by inhibiting β-catenin activation.

*N*-Ac-l-Leu-PEI delivered miR-34a *in vitro* and *in vivo* with sufficient biocompatibility and transfection efficiency. *N*-Ac-l-Leu-PEI/miR-34a delivery stabilized the anchorage teeth and promoted osteogenic differentiation *in vitro* and local alveolar bone formation *in vivo* during orthodontic force loading. The regulatory mechanism of miR-34a might be attributed to its effect on the GSK-3β target. MiR-34a is a potential target of molecular orthodontic therapy for local alveolar bone remodeling.

## MATERIALS AND METHODS

### Materials

MiR-34a agomir and its antisense oligonucleotide antagomir (miR and anti-miR control), stable negative control, inhibitor negative control, fluorescein isothiocyanate (FITC)-labeled miR-34a, U6, and SYBR Green Hairpin-it miRNAs qRT-PCR kit were purchased from GenePharma (Suzhou, China). HiPure Total RNA kits were purchased from Magen (Guangzhou, China). The PrimerScript® RT reagent kit and SYBR Green Premix Ex Taq, and the primers for β-actin, Runx2, ColI, and ALP were purchased and synthesized in Takara (Dalian, China). The sequences are listed in Table 1. *N*-Ac-l-Leu-PEI was constructed according to previous methods [22].

**Table 1:** Gene sequence

Gene	Forward primers	Reverse primers
β-actin	GGAGATTACTGCCCTGGCTCCTA	GACTCATCGTACTCCTGCTTGCTG
Runx2	CATGGCCGGGAATGATGAG	TGTGAAGACCGTTATGGTCAAAGTG
ALP	CATCGCCTATCAGCTAATGCACA	ATGAGGTCCAGGCCATCCAG
ColI	GACATGTTCAGCTTTGTGGACCTC	AGGGACCCTTAGGCCATTGTGTA
U6	CGCTTCACGAATTTGCGTGTCAT	CAAAGTGCTTACAGTGCAGGTAG
miR-34a agomir	UGGCAGUGUCUUAGCUGGUUGU	AACCAGCUAAGACACUGCCAUU
miR-34a antagomir	ACAACCAGCUAAGACACUGCCA	
miR-NC	UUCUCCGAACGUGUCACGUTT	ACGUGACACGUUCGGAGAATT
Inhibitor-NC	CAGUACUUUUGUGUAGUACAA	

### rBMSC culture

The bone stromal stem cells of Wistar rats (rBMSCs) were purchased from Cyagen (Guangdong, China). The rBMSCs were cultured in growth medium containing L-DMEM (Gibco, USA), 10% fetal bovine serum (BI, Israel), and penicillin (100 U/mL)/streptomycin (100l g/mL) (HyClone Laboratories Inc., Logan, UT). The cells were cultured at 37°C in a 5% CO_2_ incubator, and passage 3 cells were used for the following experiments.

### Gel retardation assay

The *N*-Ac-l-Leu-PEI and miR-34a were mixed in different mass ratios and incubated at room temperature for 30 min before use. The binding capacity of *N*-Ac-l-Leu-PEI with miR-34a was then evaluated by electrophoresis (80 V, 25 min) in 1.5% agarose gel with a tris-acetate-ethylenediaminetetraacetic acid buffer solution.

### Cell viability assay

The cell viability of rBMSCs transfected with *N*-Ac-l-Leu-PEI/miR-34a complexes was determined by 3-(4,5-dimethylthiazol-2-yl)-2,5-diphenyltetrazolium (MTT) assay. Third-passage rBMSCs were seeded into a 96-well plate at a density of 5.0 × 10^3^ cells/well. The medium was replaced with 100 μL serum-free L-DMEM after 24 h incubation. The control group consisted of cells cultured with medium only. Lipofectamine^2000^/miR-34a (Life, USA) or *N*-Ac-l-Leu-PEI/miR-34a complexes of varying mass ratios with a miR-34a concentration of 1 μg/mL were transfected into cells. The medium was replaced with L-DMEM containing 10% FBS after transfection for 6 h. MTT solution (20 μL of 5 mg/mL in PBS) (Sigma-Aldrich, St Louis, MO, USA) was added to each well after 24 h, and the plate was incubated for an additional 4 h. MTT was removed from the solution, 150 μL DMSO was added (Sigma-Aldrich, St Louis, MO, USA), and formazan crystals were dissolved for 10 min. The absorbance at 492 nm was obtained with a GF-M3000 microplate reader (Shandong, China). The cell viability was calculated by *A*_sample_/*A*_control_ ×100.

### Flow cytometry and fluorescence microscopy

The transfected and control cells were collected in a dark room, followed by washing and centrifuging three times before transfer into transparent glass test tubes. The indices of flow cytometry under blue light were set at 480 nm incident and 520 nm emission wavelengths. The ratio of the FAM-positive cells/total examined cells was determined quantitatively. FAM-positive cells were observed under inverted fluorescence microscope (Olympus, Japan) after 6 h transfection.

### Transfection

The *N*-Ac-l-Leu-PEI and oligonucleotide complexes were mixed at room temperature. After 30 min these complexes were transfected into rBMSCs for 6 h or locally injected into the alveolar bone of the rats indirectly.

### Mechanical force loading

The force-loading plates (7.6 × 3.6 cm^2^ and 1.2 mm thick) were prepared on the bottom surface of a 75-cm^2^ cell canted-neck culture flask. Passage 3 rBMSCs were seeded onto the center area (5 × 3.6 cm) of the force-loading plates at a density of 2 × 10^5^ cells. The force-loading plates were subjected to daily cyclic uniaxial tensile strain (0.5 Hz, 2000 με, 2 h/day) in an osteogenic differentiation medium (1 × 10^-8^ M dexamethasone, 10 mM β-glycerophosphate, and 50 μg/ml L-ascorbic acid) (Sigma-Aldrich, St Louis, MO, USA) by a four-point bending system (Sichuan University, Chengdu, China) [27, 46].

### qRT-PCR

The total RNAs from rBMSCs (after 7 days) and alveolar bone tissue (after 14 days) after mechanical force loading were isolated using a HiPure Total RNA kit according to the manufacturer's instructions. MiRNA was subjected to reverse transcription and PCR by using an SYBR Green Hairpin-it miRNAs qRT-PCR kit. The mRNAs were investigated with a PrimerScript® RT reagent kit and SYBR Green Premix Ex Taq. The PCR products were evaluated with a MxPro Mx3005P real-time PCR detection system (Agilent Technologies, Santa Clara, CA, USA). The internal controls of mRNAs and miR-34a were β-actin and U6, respectively. The cycling conditions of mRNAs were as follows: 95°C for 30 s, followed by 40 cycles of 95°C for 5 s, 55°C for 30 s, and 72°C for 1 min. The cycling conditions for miRNAs were as follows: 95°C for 3 min, followed by 40 cycles of 95°C for 12 s and 62°C for 40 s. The 2^-ΔΔCt^ method was used to calculate the relative expression levels, and the obtained values were averaged from triplicate measurements.

### Alkaline phosphatase (ALP) activity and alkaline phosphatase staining

The rBMSCs were collected and lysed using 1% Triton X-100 after mechanical force loading for 7 days. An aliquot of cells was used for the relative quantitative measurements of ALP. The total ALP activity and protein concentration were evaluated by using an alkaline phosphatase assay kit (Nanjingjiancheng, Nanjing, China) and a bicinchoninic acid protein assay kit (Beyotime Biotech Inc., Jiangsu, China). The ALP levels were normalized to the total protein content. The remaining test cells were subjected to ALP staining (Beyotime Biotech Inc., Jiangsu, China) according to the manufacturer's instructions. The stained cells in each group were photographed, and cell staining was independently repeated at least three times.

### Western blot analysis

The proteins of rBMSCs were harvested with RIPA lysis buffer and quantified with a bicinchoninic acid protein assay kit. Proteins were separated on 10% polyacrylamide gels and transferred to polyvinylidene fluoride (PVDF) membranes (Millipore, Billerica, MA, USA). These membranes were blocked with 5% BSA in TBST and incubated with the following primary antibodies: Runx2 (1:1000, ab23981, Abcam, Hong Kong), ColI (1:650, A5786, ABclonal, Boston, USA), GSK-3β (1:650, A3174), active-β-catenin (1:1000, #8480, Cell Signaling Technology, Danvers, USA), and β-actin (1:8000, AC004, ABclonal, Boston, USA). Horseradish peroxidase-conjugated anti-rabbit or anti-mouse secondary antibodies were diluted at 1:3000 and incubated at room temperature for 1 h. The signals were detected using an ECL chemiluminescence kit (7Sea biotech, Shanghai, China) with a ChemiDoc XRS+ system (Biorad, America).

### Experimental OTM rat model

All animal procedures were approved by the Animal Care and Use Committee of Jilin University. The animals were 8-week-old male Wistar rats with an average weight of 180 g that were obtained from the Animal Experiment Center of Jilin University. The rats were stochastically divided into the miR-34a agomir and miR-34a antagomir groups. The right maxillary was the experimental lateral bone, while the left maxillary was the control lateral bone. The experimental tooth movement model was established according to a previously described method [47]. The rats were anesthetized by intraperitoneal injection of pentobarbital (6 μL/g, Merck KGaA, Germany). Nickel-titanium closed-coil springs exerted a 50 N stretching force from the maxillary incisor to the maxillary first molar to move the first molar mesially. Spring retention was monitored daily to ensure stable and continuous force. There were at least 6 rats in each group.

After the tooth movement models were successfully established, the experimental rats were divided into three groups: control, miR-34a agomir, and miR-34a antagomir. Groups were treated with PBS, *N*-Ac-l-Leu-PEI/miR-34a agomir, or antagomir (2 μg oligo/lateral/time), respectively, on the experimental lateral bone by local injection for biochemical detection. The other rats were treated with *N*-Ac-l-Leu-PEI/miR-34a agomir or antagomir (2 μg oligo/lateral/time) on the experimental lateral bone by local injection. The opposite lateral bone control was treated with *N*-Ac-l-Leu-PEI/miR-NC or inhibitor-NC (with the same dosage and ratio on the left lateral bone) in the same position. Oligo injection was cyclically performed once every 3 days for 14 days.

### Histological, immunohistochemical, and micro-CT analysis

After 14 days of orthodontic force loading, we used a full automatic biochemical analyzer to detect the ALT, AST, ALP, TBIL, CREA, and BUN levels in rat ocular blood. The influence of miR-34a on hepatic and renal function in rats was assessed.

After the other rats were sacrificed, the maxilla molar-bearing segments of the alveolar bone were cut from each side and fixed overnight in 4% paraformaldehyde buffer. Micro-CT analysis was performed on fixed specimens where each sample was exposed to an X-ray with 70 kV voltage, 200 mA node current, 220 threshold value, and 300 ms exposure time. Remaining fixed specimens were decalcified in 10% ethylenediaminetetraacetic acid (EDTA) solution (pH 7.0) for 8 weeks, embedded in paraffin, and cut into 5 μm sections parallel to the mesial and distal directions of the maxillary molars.

For morphological analysis, sections were stained with hematoxylin and eosin (H&E). For immunohistochemical studies, the sections were incubated with primary antibodies against Runx2 (1:100) at 4°C overnight with an immunohistochemistry staining kit (Maixin Bio, Fuzhou, China). Image collection and staining were analyzed by Image-Pro Plus (Media Cybernetics, Bethesda, MD, USA).

### Statistical analysis

Experiments were performed on at least three individuals. Results were presented as the mean ± standard deviation and statistically analyzed with a Student's *t*-test unless noted otherwise. A two-tailed P-value <0.05 was considered statistically significant. P-values were indicated by ^*^ P<0.05, ^**^ P<0.01, and ^***^ P<0.001, and n.s., non-significant (P>0.05). Statistical analysis was conducted in SPSS version 20.0 (IBM).

## References

[1] Yin K, Hacia JG, Zhong Z, Paine M (2014). Genome-wide analysis of miRNA and mRNA transcriptomes during amelogenesis. BMC Genomics.

[2] Liu H, Lin H, Zhang L, Sun Q, Yuan G, Zhang L, Chen S, Chen Z (2013). MiR-145 and mir-143 regulate odontoblast differentiation through targeting Klf4 and Osx genes in a feedback loop. J Biol Chem.

[3] Wang C, Liao H, Cao Z (2016). Role of osterix and microRNAs in bone formation and tooth development. Med Sci Monit.

[4] Li A, Li Y, Song T, Wang F, Liu D, Lan ZP, Cheng S, Zhang CM, Wang JS, He JQ, Wang SL (2015). Identification of differentiation of microRNA expression during tooth morphogenesis in the heterodont dentition of miniature pigs, SusScrofa. BMC Dev Biol.

[5] Park MG, Kim JS, Park SY, Lee SA, Kim HJ, Kim CS, Chun HS, Park JC, Kim DK (2014). MicroRNA-27 promotes the differentiation of odontoblastic cell by targeting APC and activating Wnt/β-catenin signaling. Gene.

[6] Sehic A, Tulek A, Khuu C, Nirvani M, Sand LP, Utheim TP (2017). Regulatory roles of microRNAs in human dental tissues. Gene.

[7] Chang M, Lin H, Luo M, Han G (2015). Integrated miRNA and mRNA expression profiling of tension force-induced bone formation in periodontal ligament cells. In Vitro Cell Dev Biol Anim.

[8] Chen Y, Mohammed A, Oubaidin M, Evans CA, Zhou X, Luan X, Diekwisch TG, Atsawasuwan P (2014). Cyclic stretch and compression forces alter microRNA-29 expression of human periodontal ligament cells. Gene.

[9] Iwawak Y, Mizusawa N, Iwata T, Higaki N, Goto T, Watanabe M, Tomotake Y, Ichikawa T, Yoshimoto K (2015). MiR-494-3p induced by compressive force inhibits cell proliferation in MC3T3-E1 cells. J Biosci Bioeng.

[10] Chen N, Sui BD, Hu CH, Cao J, Zheng CX, Hou R, Yang ZK, Zhao P, Chen Q, Yang QJ, Jin Y, Jin F (2016). MicroRNA-21 contributes to orthodontic tooth movement. J Dent Res.

[11] Krzeszinski JY, Wei W, Huynh H, Jin Z, Wang X, Chang TC, Xie XJ, He L, Mangala LS, Lopez-Berestein G, Sood AK, Mendell JT, Wan Y (2014). MiR-34a blocks osteoporosis and bone metastasis by inhibiting osteoclastogenesis and Tgif2. Nature.

[12] Wan M, Gao B, Sun F, Tang Y, Ye L, Fan Y, Klein OD, Zhou X, Zheng L (2012). MicroRNA miR-34a regulates cytodifferentiation and targets multi-signaling pathways in human dental papilla cells. PLoS One.

[13] Sun F, Wan M, Xu X, Gao B, Zhou Y, Sun J, Cheng L, Klein OD, Zhou X, Zheng L (2014). Crosstalk between miR-34a and Notch signaling promotes differentiation in apical papilla stem cells (SCAPs). J Dent Res.

[14] Grilli A, Sciandra M, Terracciano M, Picci P, Scotlandi K (2015). Integrated approaches to miRNAs target definition: time-series analysis in an osteosarcoma differentiative model. BMC Med Genomics.

[15] Boussif O, Lezoualc'h F, Zanta MA, Mergny MD, Scherman D, Demeneix B, Behr JP (1995). A versatile vector for gene and oligonucleotide transfer into cells in culture and in vivo: polyethylenimine. Proc Natl Acad Sci U S A.

[16] Tian H, Xiong W, Wei J, Wang Y, Chen X, Jing X, Zhu Q (2007). Gene transfection of hyperbranched PEI grafted by hydrophobic amino acid segment PBLG. Biomaterials.

[17] Tian H, Li F, Chen J, Huang Y, Chen X (2012). N-isopropylacrylamide-modified polyethylenimines as effective gene carriers. Macromol Biosci.

[18] Dong X, Lin L, Chen J, Guo Z, Tian H, Li Y, Wei Y, Chen X (2013). A serum-tolereant hydroxyl-modified polyethylenimine as versatile carriers of pDNA/siRNA. Macromol Biosci.

[19] Mahato M, Sharma AK, Kumar P (2013). Synthesis and characterization of N-ethyl-N’-(3-dimethylaminopropyl)-guanidinyl-polyethylenimine polymers and investigation of their capability to deliver DNA and siRNA in mammalian cells. Colloids Surf B Biointerfaces.

[20] Fischer D, Li Y, Ahlemeyer B, Krieqlstein J, Kissel T (2003). *In vitro* cytotoxicity testing of polycations: influence of polymer structure on cell viability and hemolysis. Biomaterials.

[21] Funhoff AM, van Nostrum CF, Lok MC, Fretz MM, Crommelin DJ, Hennink WE (2004). Poly (3-guanidinopropyl methacrylate): a novel cationic polymer for gene delivery. Bioconjug Chem.

[22] Li Z, Zhang L, Li Q (2015). Induction of apoptosis on cancers cells through N-acetyl-L-leucine-modified polyethylenimine-mediated p53 gene delivery. Colloids Surf B Biointerfaces.

[23] Xing Z, Gao S, Duan Y, Hao H, Li L, Yang Y, Li QS (2015). Delivery of DNAzyme targeting aurora kinase Ato inhibit the proliferation and migration of human prostate cancer. Int J Nanomedicine.

[24] Krishnan V, Davidovitch Z (2006). Cellular, molecular, and tissue-level reactions to orthodontic force. Am J Orthod Dentofacial Orthop.

[25] Wise GE, King GJ (2008). Mechanisms of tooth eruption and orthodontic tooth movement. J Dent Res.

[26] Agostini M, Knight RA (2014). miR-34: from bench to bedside. Oncotarget.

[27] Li J, Hu C, Han L, Liu L, Jing W, Tang W, Tian W, Long J (2015). MiR-154-5p regulates osteogenic differentiation of adipose-derived mesenchymal stem cells under tensile stress through the Wnt/PCP pathway by targeting Wnt11. Bone.

[28] Beg MS, Brenner AJ, Sachdev J, Borad M, Kang YK, Stoudemire J, Smith S, Bader AG, Kim S, Hong DS (2017). Phase I study of MRX34, a liposomal miR-34a mimic, administered twice weekly in patients with advanced solid tumors. Invest New Drugs.

[29] Case N, Ma M, Sen B, Xie Z, Gross TS, Rubin J (2008). β-catenin levels influence rapid mechanical responses in osteoblasts. J Biol Chem.

[30] Sen B, Xie ZH, Case N, Ma M, Rubin C, Tubin J (2008). Mechanical strain inhibits adipogenesis in mesenchymal stem cells by stimulating a durable β-catenin signal. Endocrinology.

[31] Hens JR, Wilson KM, Dann P, Chen X, Horowitz MC, Wysolmerski JJ (2005). TOPGAL mice show that the canonical Wnt signaling pathway is active during bone development and growth and is activated by mechanical loading in vitro. J Bone Miner Res.

[32] Sawakami K, Robling AG, Ai M, Pitner ND, Liu D, Warden SJ, Li J, Maye P, Rowe DW, Duncan RL, Warman ML, Turner CH (2006). The Wnt co-receptor LRP5 is essential for skeletal mechanotransduction but not for anabolic bone response to parathyroid hormone treatment. J Biol Chem.

[33] Shuqin L, Shan Y, Aishu R, Hongwei D (2015). [Investigation of Wnt/β-catenin signaling pathway on regulation of Runx2 in cementoblasts under mechanical stress in vitro]. [Article in Chinese]. Hua Xi Kou Qiang Yi Xue Za Zhi.

[34] Owan I, Burr DB, Turner CH, Qiu J, Tu Y, Onyia JE, Duncan RL (1997). Mechanotransduction in bone: osteoblasts are more responsive to fluid forces than mechanical strain. Am J Physiol.

[35] Yan YX, Gong YW, Guo Y, Lv Q, Guo C, Zhuang Y, Zhang Y, Li R, Zhang XZ (2012). Mechanical strain regulates osteoblast proliferation through integrin-mediated ERK activation. PLoS One.

[36] Mai ZH, Peng ZL, Zhang JL, Chen L, Liang HY, Cai B, Ai H (2013). MiRNA expression profile during fluid shear stress-induced osteogenic differentiation in MC3T3-E1 cells. Chin Med J.

[37] Kang H, Chen H, Huang P, Qi J, Qian N, Deng L, Guo L (2016). Glucocorticoids impair bone formation of bone marrow stromal stem cells by reciprocally regulating microRNA-34a-5p. Osteoporos Int.

[38] Chen L, Holmstrom K, Qiu W, Ditzel N, Shi K, Hokland L, Kassem M (2014). MicroRNA-34a inhibits osteoblast differentiation and in vivo bone formation of human stromal stem cells. Stem Cells.

[39] Wei J, Shi Y, Zheng L, Zhou B, Inose H, Wang J, Guo XE, Grosschedl R, Karsenty G (2012). miR-34s inhibit osteoblast proliferation and differentiation in the mouse by targeting SATB2. J Cell Biol.

[40] Tamura M, Uyama M, Sugiyama Y, Sato M (2013). Canonical Wnt signaling activates miR-34 expression during osteoblastic differentiation. Mol Med Rep.

[41] Fan C, Jia L, Zheng Y, Jin C, Liu Y, Liu H, Zhou Y (2016). MiR-34a promotes osteogenic differentiation of human adipose-derived stem cells via the RBP2/NOTCH1/CYCLIN D1 coregulatory network. Stem Cell Reports.

[42] Armstrong VJ, Muzylak M, Sunters A, Zaman G, Saxon LK, Price JS, Lanyon LE (2007). Wnt/β-catenin signaling is a component of osteoblastic bone cell early responses to load-bearing and requires estrogen receptorα. J Biol Chem.

[43] Robinson JA, Chatterjee-Kishore M, Yaworsky PJ, Cullen DM, Zhao W, Li C, Kharode Y, Sauter L, Babij P, Brown EL, Hill AA, Akhter MP, Johnson ML (2006). Wnt/β-catenin signaling is a normal physiological response to mechanical loading in bone. J Biol Chem.

[44] Yuan HF, Von Roemeling C, Gao HD, Zhang J, Guo CA, Yan ZQ (2015). Analysis of altered microRNA expression profile in the reparative interface of the femoral head with osteonecrosis. Exp Mol Pathol.

[45] Kim NH, Cha YH, Kang SE, Lee Y, Lee I, Cha SY, Ryu JK, Na JM, Park C, Yoo HG, Park GJ, Yook JI, Kim HS (2013). p53 regulates nuclear GSK-3 levels through miR-34-mediated Axin2 suppression in colorectal cancer cells. Cell Cycle.

[46] Liu J, Liu T, Zheng Y, Zhao Z, Liu Y, Cheng H, Luo S, Chen Y (2006). Early responses of osteoblast-like cells to different mechanical signals through various signaling pathways. Biochem Biophys Res Commun.

[47] King GJ, Keeling SD, McCoy EA, Ward TH (1991). Measuring dental drift and orthodontic tooth movement in response to various initial forces in adult rat. Am J Orthod Dentofacial Orthop.

